# Supplementary material for: Multidimensional Predictors of Cancer-Related Fatigue Based on the Predisposing, Precipitating, and Perpetuating (3P) Model: A Systematic Review
Source: Cancers (Basel). 2023 Dec 17;15(24):5879. doi: 10.3390/cancers15245879 (PMC10741552; doi:10.3390/cancers15245879)
Supplement: Supplementary file 1 [file cancers-15-05879-s001.zip › cancers-2631475-supplementary.pdf]

This is a specific search strategy for PubMed databases, and a combination of subject terms and accessible terms approach is also applicable to other databases.

(((((fatigue[MeSH Terms]) OR (Fatigue Syndrome, Chronic[MeSH Terms])) OR (fatigue)) AND (((Neoplasms[MeSH Terms]) OR (cancer)) OR (Neoplasms))) OR (("cancer related fatigue") OR (CRF))) AND (((((biomarkers[MeSH Terms]) OR (risk factors[MeSH Terms])) OR ("Predictive factors")) OR (Predictors)) OR (predictive)) **6,819**

((("fatigue"[MeSH Terms] OR "fatigue syndrome, chronic"[MeSH Terms] OR ("fatiguability"[All Fields] OR "fatiguable"[All Fields] OR "fatigue"[MeSH Terms] OR "fatigue"[All Fields] OR "fatigued"[All Fields] OR "fatigues"[All Fields] OR "fatiguing"[All Fields] OR "fatigueability"[All Fields])) AND ("neoplasms"[MeSH Terms] OR ("cancer s"[All Fields] OR "cancerated"[All Fields] OR "canceration"[All Fields] OR "cancerization"[All Fields] OR "cancerized"[All Fields] OR "cancerous"[All Fields] OR "neoplasms"[MeSH Terms] OR "neoplasms"[All Fields] OR "cancer"[All Fields] OR "cancers"[All Fields]) OR ("neoplasm s"[All Fields] OR "neoplasms"[MeSH Terms] OR "neoplasms"[All Fields] OR "neoplasm"[All Fields]))) OR ("cancer related fatigue"[All Fields] OR "CRF"[All Fields])) AND ("biomarkers"[MeSH Terms] OR "risk factors"[MeSH Terms] OR "Predictive factors"[All Fields] OR ("predictor"[All Fields] OR "predictors"[All Fields]) OR ("predict"[All Fields] OR "predictabilities"[All Fields] OR "predictability"[All Fields] OR "predictable"[All Fields] OR "predictably"[All Fields] OR "predicted"[All Fields] OR "predicting"[All Fields] OR "prediction"[All Fields] OR "predictions"[All Fields] OR "predictive"[All Fields] OR "predictively"[All Fields] OR "predictiveness"[All Fields] OR "predictives"[All Fields] OR "predictivities"[All Fields] OR "predictivity"[All Fields] OR "predicts"[All Fields]))
